# Space exploration and economic growth: New issues and horizons

**DOI:** 10.1073/pnas.2221341120

**Published:** 2023-10-16

**Authors:** Luisa Corrado, Maureen Cropper, Akhil Rao

**Affiliations:** ^a^Department of Economics and Finance, University of Tor Rome Vergata, Rome 00133, Italy; ^b^Department of Economics, University of Maryland, College Park, MD 20742; ^c^Department of Economics, Middlebury College, Middlebury, VT 05753

Space has been a critical factor in the growth and development of modern economies (1–3). Positioning systems such as Global Positioning System (GPS) have significantly impacted shipping and trade (4). Remote sensing and telecommunications have enabled rapid response to natural disasters (5) and better estimates of economic activity (6, 7). Additionally, space-based technologies have improved arms control treaties and enabled better monitoring of armed conflicts (8–10).

Although the average individual rarely interacts directly with space, almost all aspects of modern economies connect to it. These trends have become particularly prominent over the past few decades, as technology and policy shifts such as reusable rockets, greater computing power, and new contracting mechanisms have led to lower launch prices and increased commercial interest in space (11). These changes are not limited to a few high-income economies—while the United States and China are currently launching the most payloads to space, the total number of countries with payloads in space has never been higher. Figs. 1 and 2 plot the number of countries launching payloads to space and average launch prices over time.

**Fig. 1.**
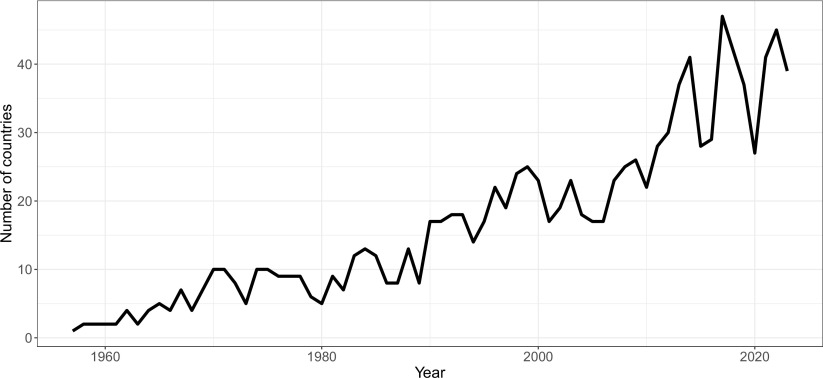
Number of countries launching payloads to space each year. Sources: https://www.space-track.org/ and authors’ calculations.

**Fig. 2.**
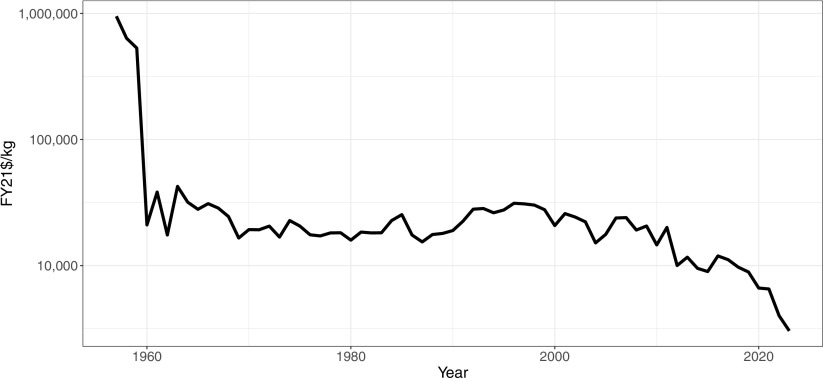
Price of launching one kilogram of payload mass to LEO as part of a dedicated launch (FY21$/kg denotes estimates using dollar values in fiscal year 2021). LEO is the region between 100 and 2,000 km above mean sea level. Source: authors’ calculations (12).

As an economic environment, space is unique. The Outer Space Treaty of 1967 is the predominant international legal framework governing human activity in space. This treaty states that outer space is not subject to national appropriation by claim of sovereignty, complicating the process of establishing property rights in space. The current legal regime poses challenges to economic development in space (13–15), as do geopolitical and military considerations, and significant state involvement in space-related industries. While an expansive body of research has considered the use of various principles of commons governance in managing space activities and resources without establishing property rights (16–18), economists have long recognized the importance of property rights for efficient resource allocation (19, 20).

This Special Feature on “Space Exploration: Economics, Technologies, and Policies” explores key issues in space economics, focusing on the roles of states and firms in technology development, resource management, and economic growth. First, it assesses the implications of significant capital investments in space technologies, both historically and in their potential to combat secular stagnation. Second, it examines the role of the public sector in supporting and regulating the expanding space economy, both identifying promising new institutional structures and characterizing and quantifying their benefits. Finally, it considers the implications of more intensive orbit use and extraction of space mineral resources, outlining salient environmental trade-offs and policy catalysts.

## Historical Perspective on Space Exploration and Economic Growth

Prevailing views emphasize the huge role of government funding in promoting space exploration, against which the modern rise of private funding is an exception. But historical analysis reveals that private funding for space exploration, particularly in the United States, is more a norm than a recent anomaly (21).

The belief that the early years of the “Space Race” led to substantial economic growth in the United States is also widespread (22), but empirical evidence is scant and rarely addresses the question: What is the economic impact of space investment? Ref. 23 in this Special Feature examines the economic effects of space-related activities on Earth. The authors empirically assess the effects of space missions in the United States from the 1960s to the present day in an economy where technologies developed in the space sector can affect other sectors. For example, discoveries generated by increase in space activity, such as new tracking systems (e.g., GPS) or more compact hardware (e.g., laptops), can also boost productivity in non-space-related sectors, driving economic output (measured by Gross Domestic Product, GDP) to a higher growth trajectory.

Their main finding is that space activities provide positive spillovers to the economy with different intensities over time. These intensities reached their highest values between the end of the 1960s and the beginning of the 1980s and their lowest values in the 2000s. They consider an exercise that increases space-related production by the same amount under the high and low spillover scenarios and find that the transmission effects on output growth are more than double when associated with the high spillover scenario of the early decades of space activity. Specifically, space sector activity in the 1960s and 1970s had large positive impacts on GDP growth, increasing real GDP by 2.2% on average after 20 y. By contrast, space activity since the 1980s—when public space investment in the United States slowly waned as tasks were outsourced to private industry or simply no longer conducted—has had much smaller 20-y impacts on real GDP, on the order of 0.9%.

These results provide lessons for existing and emerging space powers as they look to the historical record for guidance. Positive growth spillovers from space spending may be particularly attractive to policymakers in high-income economies to counterbalance stagnant growth and to use as a tool to fight deep downturns. However, it is not yet clear whether and how the largest effects of the early decades of space activity can be replicated by new space spending. Further research is needed on public space spending, the structure of the sector, and its role in economies at large.

## Challenges and New Institutions

The roles of the public and private sectors are rapidly changing in the modern era, with governments paving the way for private corporations to build large, coordinated systems of satellites. These changes are spurred by major technological developments (e.g., reusable rocket boosters and cubesats^*^), policy changes (e.g., greater use of commercial contracting), and the rise of private funding by wealthy individuals (e.g., Elon Musk and Jeff Bezos) (11). Against this backdrop, it is worth asking a fundamental question: What should the role of the public sector be in ensuring that space exploration has positive impacts on economic growth? Refs. 24 and 25 in this Special Feature offer perspectives on this question. Two challenges, in particular, stand in the way of harnessing space activity to boost economic growth: research and development spending and policies governing the use of space.

Concerning the first challenge, private companies often prioritize research efforts that produce profitable projects in the short run. These findings may be kept secret, particularly when they are expected to yield competitive advantages (26, 27). In contrast, public sector research efforts focus on the development and widespread dissemination of public knowledge, especially knowledge that can be used by many firms. Moreover, private companies are willing to take more risk and move faster than government bureaucracies. The first challenge is, therefore, to find ways for public policy to structure the R&D environment to maximize R&D production and diffusion.

In addressing this question, ref. 24 argues that Public-Private Research and Development Partnerships (PPRDPs) between government, private industry, and research universities can effectively link the private sector to public sources of financial and intellectual capital. They can also coordinate private innovation activities and intellectual property rights to maximize their impact. For example, a PPRDP may connect a private company working on a biotechnological activity with research and development (R&D) funds stewarded by NASA and with scientists and students from research universities. PPRDPs can help companies colocate and create regional innovation clusters to reduce the costs of knowledge sharing, thus creating more efficient labor markets for specialized workers and firms (28, 29). However, the allocation of control rights, which involve decisions in the face of contingencies not fully specified ex ante, is a central issue in implementing effective PPRDPs for space R&D. To address this issue, ref. 24 proposes a decentralized autonomous organization framework. This utilizes smart contracts—software programs that are executed automatically when prespecified “if-then” conditions are met—to reduce counterparty risk and dependency on trust among members. This innovative approach to PPRDPs can help to bridge the gap between public sector organizations, private firms, and research universities, ultimately leading to more efficient and effective R&D.

The second challenge lies in the use of space and the market structure for satellite services. Most space activity is concentrated in the orbital space around Earth, particularly in low-Earth orbit (LEO), the region between 100 and 2,000 km above mean sea level. New launch technologies and in-space R&D activities are likely to be spurred by the use of orbital space. The lack of orbital property rights due to the Outer Space Treaty will lead to costly misallocation of orbital space and overproduction of orbital debris and collision risk (14, 30–32). However, it is unclear how these issues will be dealt with in the era of large satellite constellations—coordinated fleets of hundreds or thousands of satellites operated by individual firms—providing global telecommunications services. These systems feature substantial economies of scale (33), which means that very few firms are likely to successfully operate them. The operators of these systems will likely target markets that are poorly served by existing terrestrial systems, so they are unlikely to face significant competition from non-space actors. Such imperfect or oligopolistic competition will push constellation operators to overly restrict system sizes to minimize costs and reduce pricing pressure. On the other hand, neglecting the environmental damages of maintaining large numbers of satellites [e.g., the effects of launch activities on the atmosphere (34, 35), the deleterious effects on astronomy (36), and overproduction of orbital debris] will lead operators to choose larger system sizes than would be socially optimal. The challenge is determining how public policy should regulate these systems to ensure that their economic net benefits are maximized.

Ref. 25 studies the policy implications of market structure in the context of duopoly constellations competing for profits and orbital space. The authors find that the combination of imperfect competition and profitability of minimizing collision avoidance will produce a highly unequal distribution of constellation service qualities. The first mover will build a large system in the most valuable region to provide high-quality service to the majority of the market, while the follower will build a much smaller system at a higher altitude and provide lower-quality service to a small segment of the market. The optimal “public utility system” that maximizes economic welfare involves two large constellations placed closer together at lower altitudes, each offering much more comparable service qualities to approximately half the market. These public utility constellations provide more equitable service to the entire market and greater economic net benefits.

While there is substantial uncertainty over the scope and magnitude of environmental damages caused by orbit use, it seems clear that the damages are nonzero. Therefore, the authors examine how varying the magnitude of these damages affects optimal policy design. They find that at low levels of environmental damages, economic net benefits are maximized by maintaining more satellites in orbit than the duopoly would, while at high levels of environmental damages, economic net benefits are maximized by maintaining fewer satellites in orbit than the duopoly would. These findings emphasize the importance of studying the implications of competitive behavior in space for space resource use, quantifying the environmental and social costs of different ways of allocating space resources, and developing effective governance and regulatory models to maximize the global economic net benefits of orbit use.

## Looking Ahead: Space as a Catalyst for Sustained Economic Growth

Looking ahead, generating sustained economic growth from space will likely require significant levels of capital investment. How might investments in space exploration interact with slowing growth on Earth, particularly in high-income economies? And how might the ability to access mineral resources in space affect growth on Earth, particularly in the face of ongoing environmental degradation? The final two articles in this Special Feature address these questions.

Ref. 37 offers a unique solution to the problems of “secular stagnation”— a state of self-fulfilling, persistently sluggish economic growth—that plagues modern high-income economies (38–40). Modern theories of secular stagnation emphasize the need to sharply increase both aggregate demand and aggregate supply, e.g., through increased capital investment or growth in productivity or population. Achieving these goals in the modern era, characterized by near-zero real interest rates, declining productivity and innovation, and declining fertility in high-income economies, is challenging. On the demand side, if the United States returns to its historical peak levels of public-sector investment in space—as a share of federal government outlays or GDP—it would directly add around 1.5 to 3.0 trillion to demand over the next two decades. On the supply side, long-established theories of innovation have emphasized the role of “frontiers” as generators of dynamism and productivity growth. Harnessing the positive growth effects of the new space environment, embarking on risky and productive ventures, and establishing new habitats sustainably are critical. The potential of space as a large-scale project to reinvigorate economic growth and improve human well-being is unmatched and merits further study.

Mineral resources naturally sit at the intersection of space exploration and economic growth. The popular press frequently cites the large abundances of minerals like platinum and cobalt in space, often valuing them at current prices. Although economic research points to more complex price dynamics and more muted valuations (41), the question remains: How would space mining affect economic growth on Earth? Ref. 42 studies these questions against the backdrop of the ongoing clean energy transition on Earth. The clean energy transition could lead to substantial increases in the demand for certain critical minerals, but increased mining activity required to meet these demands will also increase environmental degradation on Earth. Despite declines in launch costs, investment in space mining will likely be costly. Ref. 42 models this trade-off and identifies characteristics of optimal space mining transition paths. The authors find that a transition of mining from Earth to space could allow for continued growth of metal use on Earth while limiting environmental and social costs. The optimal trajectory of investment initially involves increasing investment in Earth’s mining capital due to the cumulative history of R&D and greater initial productivity. After a phase of R&D to increase the productivity of space investment, the optimal trajectory features a sharp reallocation of investment from Earth to space mining capital, with metals output from space eventually surpassing Earth‘s output. The reallocation of investment is driven by the need to constrain greenhouse gas emissions on Earth, with tighter carbon ceilings spurring more rapid transitions to space mining.

## Open Questions and Next Steps

This Special Feature deals with several important topics—the economic impact of space investment, innovation-inducing organizational design, competition and optimal use of common pool resources, the potential for cleaner green transitions, and answers to secular stagnation—but it is not the last word on any of them. Rather, by exploring how expanding activities in space have contributed, are contributing, and can contribute to economic growth, these articles are meant to draw attention to the field and identify new and fruitful research directions. In addition to the questions identified throughout this Special Feature, we highlight four areas of study that can have a significant impact on the economics of space exploration and economic growth.

First, it is important to remember that the budgetary investment related to space exploration has historically been linked to military objectives, such as the Cold War, which focused on geopolitical and military objectives rather than economics (8, 21). That this period of US government spending also produced the largest positive economic growth spillovers is cause for further detailed study (23). Similar motives are sometimes cited in relation to the current boom in satellite constellations (43) and national interest in space resource mining (44). They also shape constraints on international trade and cooperation in space (45–47). These issues will pose novel challenges to policy frameworks for managing competition, governing resource use, and fostering innovation.

Second, while there is great potential for economic growth from space, continuing space development along its current trajectory will also create market failures that limit this potential. These include misallocation of orbital space and overproduction of orbital debris (25), as well as underprovision of public goods such as planetary protection against asteroids and backward contamination of Earth. Solving these global risks poses global collective action problems, and their solution will require international coordination (48). While orbital space management has received some attention, there is much less research on the types of policy and economic structures that can efficiently support planetary protection.

Third, although space exploration has the potential to boost future economic growth and scientific progress (24, 37), it comes at a time of increasing global income and wealth inequality (49) and unprecedented climate-driven disruption (50–53). While the transition to space mining may help meet ambitious climate goals without reducing economic growth (42), this transition may disrupt production patterns, potentially impacting workers in mining-dependent countries. Research on mechanisms to encourage innovation and dynamism in space while ensuring equitable economic benefits can help identify positive pathways for space exploration.

Finally, effective policies and reliable predictions in the space sector demand comprehensive, high-frequency data. Such specialized statistics are currently scarce, often compelling analysts to rely on industry or government sources not specifically designed for studying the space sector (14, 23). These sources often lack details on firms' locations, production patterns, environmental costs, input factor uses, capital structures, and supply chains. The collection and dissemination of such detailed economic data could have significant positive effects (54) by providing information to make accurate predictions and formulate detailed policies (55). While there are promising steps in this direction (56), space data infrastructure requires further improvement.

If managed well, the exploration and utilization of space could present unprecedented opportunities for economic development and sustainability. The articles of this Special Feature emphasize this potential and highlight the need for informed policy-making and international cooperation to govern human expansion into the cosmos.

## Data Availability

Code and data used to generate Figs. 1 and 2 have been deposited in the Middlebury Institutional Repository (12).

## Footnotes

^*^“Cubesat” is a class of miniature satellites of cubic form. Typically using commercial components for their electronics and construction, the smallest standard cubesats have a mass of no more than 2 kg each and a size not exceeding 10 cm^3^.
